# Supplementary figures and images for: Phospholipase A2 from krait Bungarus fasciatus venom induces human cancer cell death in vitro
Source: PeerJ. 2019 Dec 3;7:e8055. doi: 10.7717/peerj.8055 (PMC6896944; doi:10.7717/peerj.8055)

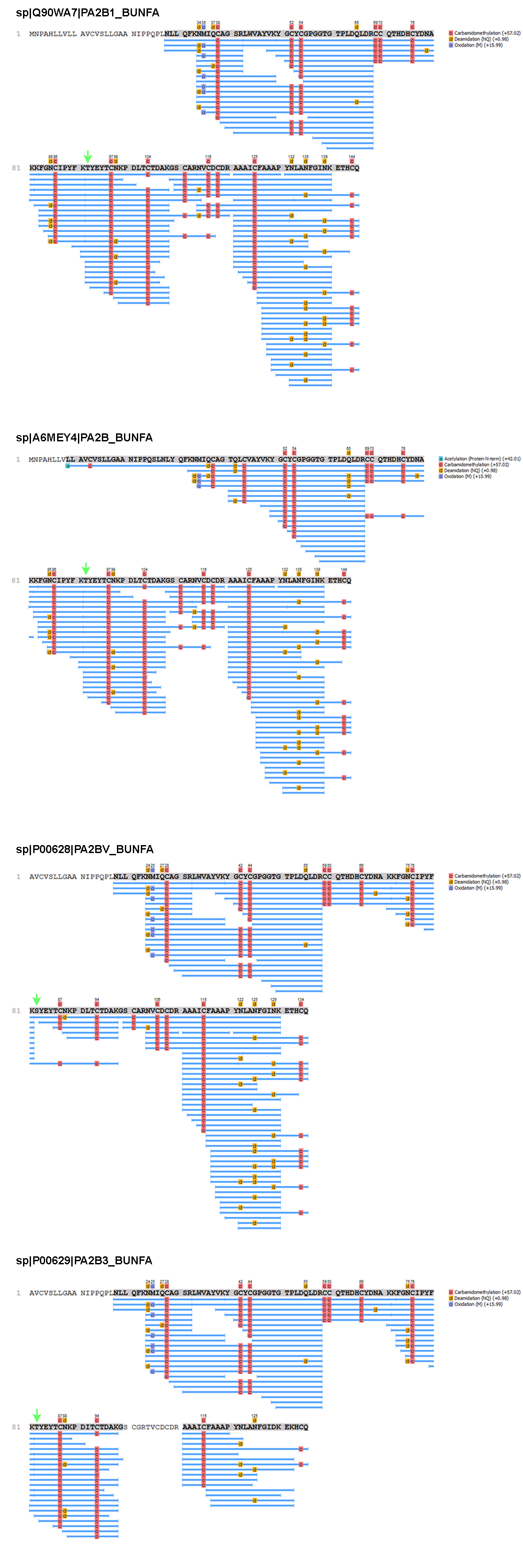

Supplement: Supplemental Information 1 — The first four sequences indentified in database and possessing the highest scores are shown. [file peerj-07-8055-s001.png]
