# Supplementary material for: Phospholipase A2 from krait Bungarus fasciatus venom induces human cancer cell death in vitro
Source: PeerJ. 2019 Dec 3;7:e8055. doi: 10.7717/peerj.8055 (PMC6896944; doi:10.7717/peerj.8055)
Supplement: Supplemental Information 4 [file peerj-07-8055-s004.docx]

**Table 3S**

Cytotoxicity of snake venom phospholipases A2 to different cancer cell lines.

| Phospholipase A2; Snake species | Cell line | Activity | Reference |
| --- | --- | --- | --- |
| Crotoxin; *Crotalus durissus* | Murine erythroleukemia (MEL) cells, clone DS-19 | The 50% inhibitory concentration (IC50) - approximately 0.1-0.2 µM (3.0-5.0 µg/ml) | Corin et al., 1993 |
|  | Chronic myeloid leukemia cell line K562 | The IC50 was 32.73 μg/ml after 48 h of treatment. At 72 h of threatment with 50 μg/ml - the inhibition rate 85.9% | Yan et al., 2006 |
|  | MCF-7 | At 72 h of treatment with 50 μg/mL - the inhibitory rate 33.8%; with 100 μg/mL - the inhibitory rate 57.0% | Yan et al., 2007 |
|  | Human lung adenocarcinoma A549 cells | Treatment for 48 h; IC50 78 μg/mL | Ye et al., 2011 |
| BJ-PLA2-I; *Bothrops jararaca* | Human leukemia cell line HL-60 | Viabilities between 70 and 80% at 40-120 µg/ml | Cedro et al., 2018 |
| BthA-I-PLA2; *B. jararacussu* | Leukemic cells Jurkat; human breast tumor cells SK-BR-3; Ehrlich ascites tumor (EAT) cells | 100 μg/mL;  Jurkat viability 50%;  SK-BR-3 viability 30%;  EAT cells viability 80% | Roberto et al., 2004 |
| BmooTX-I; *B. moojeni* | Jurkat cells | 100 μg/mL; viability 50% | Santos-Filho et al., 2008 |
| MTX-I; *B. brazili* | Jurkat cells | 100 μg/mL; viability 40% | Costa et al., 2008 |
| MjTX-I, *B. moojeni* | Chronic myeloid leukemia cell lines K562-S and K562-R | The IC50 values for K562-S and K562-R cells were 257 μg/mL and 191 μg/mL, respectively | Benati RB, et al., 2018 |
| BnSP-6; *B. pauloensis* | Human breast cancer cells MDA-MB-231 | 12.5–100 µg/mL; damaging about 10 to 45% of cells | Azevedo et al., 2016 |
| VBBPLA2; *Vipera berus berus* | Chronic myeloid leukemia cells K-562 | At 48 h of treatment with 100 μg/mL (7.23 μM) the viability reduced to 20% | Samel et al., 2013 |
| Pllans-II, an acidic monomeric  Asp49-PLA2 from *Porthidium lansbergii lansbergii* | human breast cancer cell MCF7; cervix adenocarcinoma cell line HeLa | At 24 h of treatment with 100 μg/mL;  HeLa cells viability to 45%;  MCF7 cells viability about 80% | Jiménez-Charris et al., 2019 |
| Recombinant basic PLA(2) (rSSBPLA(2)); *Lapemis hardwickii* | Human myeloid leukemia cells HL-60; human neuroblastoma cells SK-N-SH; human gastric cancer cells MGC-803 | IC50 for HL60 - 45.3 µg/ml; SK-N-SH - 57.12 µg/ml; MGC-803 - 69.3 µg/ml | Liang et al., 2005 |
| Nigexine; *Naja nigricollis* | Mouse neuroblastoma cells C-13 T; human myeloid leukemia cells HL-60 | IC50 for C-13T – 2.9 µM (38.6 µg/ml); HL-60 – 3.1 µM (41.2 µg/ml) | Chwetzoff et al., 1989 |

Azevedo FV, Lopes DS, Cirilo Gimenes SN, Achê DC, Vecchi L, Alves PT, Guimarães Dde O, Rodrigues RS, Goulart LR, Rodrigues Vde M, Yoneyama KA. 2016. Human breast cancer cell death induced by BnSP-6, a Lys-49 PLA₂ homologue from Bothrops pauloensis venom. *International Journal of Biological Macromolecules* . 82:671-677. DOI: 10.1016/j.ijbiomac.2015.10.080.

Benati RB, Costa TR, Cacemiro MDC, Sampaio SV, de Castro FA, Burin SM. 2018. Cytotoxic and pro-apoptotic action of MjTX-I, a phospholipase A2 isolated from Bothrops moojeni snake venom, towards leukemic cells. *The Journal of Venomous Animals and Toxins Including Tropical Diseases* 24:40. DOI: 10.1186/s40409-018-0180-9.

Cedro RCA, Menaldo DL, Costa TR, Zoccal KF, Sartim MA, Santos-Filho NA, Faccioli LH, Sampaio SV. 2018. Cytotoxic and inflammatory potential of a phospholipase A2 from Bothrops jararaca snake venom. *The Journal of Venomous Animals and Toxins Including Tropical Diseases* 24:33. DOI: 10.1186/s40409-018-0170-y.

Corin RE, Viskatis LJ, Vidal JC, Etcheverry MA. 1993. Cytotoxicity of crotoxin on murine erythroleukemia cells in vitro. *Investigational new drugs* 11:11-15.

Jiménez-Charris E, Lopes DS, Gimenes SNC, Teixeira SC, Montealegre-Sánchez L, Solano-Redondo L, Fierro-Pérez L, Rodrigues Ávila VM. 2019. Antitumor potential of Pllans-II, an acidic Asp49-PLA2 from Porthidium lansbergii lansbergii snake venom on human cervical carcinoma HeLa cells. *International Journal of Biological Macromolecules* 22:1053-1061. DOI: 10.1016/j.ijbiomac.2018.09.053.

Roberto PG, Kashima S, Marcussi S, Pereira JO, Astolfi-Filho S, Nomizo A, Giglio JR, Fontes MR, Soares AM, França SC. 2004. Cloning and identification of a complete cDNA coding for a bactericidal and antitumoral acidic phospholipase A2 from Bothrops jararacussu venom. *Protein Journal* 23:273-285.

Samel M, Vija H, Kurvet I, Künnis-Beres K, Trummal K, Subbi J, Kahru A, Siigur J. 2013. Interactions of PLA2-s from Vipera lebetina, Vipera berus berus and Naja naja oxiana venom with platelets, bacterial and cancer cells. *Toxins (Basel)* 5:203-223. DOI: 10.3390/toxins5020203.

Santos-Filho NA, Silveira LB, Oliveira CZ, Bernardes CP, Menaldo DL, Fuly AL, Arantes EC, Sampaio SV, Mamede CC, Beletti ME, de Oliveira F, Soares AM. 2008. A new acidic myotoxic, anti-platelet and prostaglandin I2 inductor phospholipase A2 isolated from Bothrops moojeni snake venom. *Toxicon* 52:908-917. DOI: 10.1016/j.toxicon.2008.08.020.

Yan CH, Liang ZQ, Gu ZL, Yang YP, Reid P, Qin ZH. 2006. Contributions of autophagic and apoptotic mechanisms to CrTX-induced death of K562 cells. *Toxicon* 47:521-530. DOI: 10.1016/j.toxicon.2006.01.010

Yan CH, Yang YP, Qin ZH, Gu ZL, Reid P, Liang ZQ. 2007. Autophagy is involved in cytotoxic effects of crotoxin in human breast cancer cell line MCF-7 cells*. Acta Pharmacologica Sinica* 28:540-548. DOI: 10.1111/j.1745-7254.2007.00530.x.

Ye B, Xie Y, Qin ZH, Wu JC, Han R, He JK. 2011. Anti-tumor activity of CrTX in human lung adenocarcinoma cell line A549. *Acta Pharmacologica Sinica* 32:1397-1401. DOI: 10.1038/aps.2011.116.
